# Usefulness of the Electrocardiogram in a Patient Presenting with Right-Sided Pneumothorax and Presyncope

**DOI:** 10.3390/diagnostics11061069

**Published:** 2021-06-10

**Authors:** Lavinia Maria Florescu, Călina-Patricia Țentea, Csilla-Andrea Eötvös, Roxana-Daiana Lazar, Iulia-Georgiana Zehan, Wissam Sabha, Sorin Pop, Doina Adina Todea, Dan Blendea

**Affiliations:** 1 Cluj County Emergency Hospital, 400020 Cluj-Napoca, Romania; lavinia.florescu94@yahoo.ro (L.M.F.); tenteapatricia@yahoo.it (C.-P.Ț.); daiana.pocol@yahoo.com (R.-D.L.); iuliazehan@gmail.com (I.-G.Z.); popsorin98@gmail.com (S.P.); 2 Faculty of Medicine, University of Medicine and Pharmacy “Iuliu Hatieganu”, 400000 Cluj-Napoca, Romania; csilla.andrea18@gmail.com (C.-A.E.); doina_adina@yahoo.com (D.A.T.); 3 “Niculae Stancioiu” Heart Institute, 400020 Cluj-Napoca, Romania; 4 “Leon Daniello” Clinical Hospital of Pulmonology, 400020 Cluj-Napoca, Romania; wisssiii@gmail.com

**Keywords:** electrocardiogram, pneumothorax, syncope, case report

## Abstract

We present the case of a 71-year-old man with history of smoking, pulmonary emphysema, hypertension, multivessel coronary artery disease and prior coronary artery bypass graft surgery who presented with spontaneous right-sided pneumothorax associated with phasic changes of the QRS amplitude on the electrocardiogram. While several case reports have described QRS amplitude changes associated with left-sided pneumothorax, reports of phasic ECG changes in right-sided pneumothorax are exceedingly rare. Such changes, when present in a patient with sudden onset chest pain and dyspnea, should prompt a diagnostic workup for possible pneumothorax.

## 1. Introduction

Pneumothorax (PTX) has been estimated to occur spontaneously with an incidence of 14/100,000 in men and 3/100,000 in women [1,2]. Risk factors include smoking, previous PTX, and male sex [2,3]. Iatrogenic PTX can occur in approximately 1.9% of percutaneous thoracic needle biopsies and 1.3% of cardiac implantable device procedures [4,5].

Presenting symptoms are usually sudden onset chest pain and dyspnea, and the diagnosis can be confirmed by chest X-ray, lung ultrasound, or computed tomography, all of which can accurately detect the presence of air in the pleural cavity [6]. However, in a significant number of cases, the presentation is nonspecific and some of these tests may be delayed or not performed at all. Irrespective of the specificity of the clinical presentation, most patients with chest pain will have an electrocardiogram (ECG) upon presentation. Therefore, it is worth exploring the different ECG patterns that occur in patients with PTX, like the one we are presenting.

ECG abnormalities in PTX have rarely been reported. Most of these reports referred to patients with large, left-sided PTX and the ECG findings were relatively nonspecific and included low voltage QRS complexes, PR segment shifts, ST segment shifts, inverted T waves, loss of R waves in the precordial leads, and right axis deviation. Another ECG change, which seems more specific to PTX, is represented by phasic ECG changes in QRS amplitude [7,8,9,10,11,12,13,14].

Reports in the literature of ECG changes in right-sided pneumothoraces like the one we are presenting are exceedingly rare [15].

## 2. Case Presentation

### 2.1. Patient Information and Timeline

A 71-year-old man with a history of smoking, severe pulmonary emphysema, hypertension, multivessel coronary artery disease, prior coronary artery bypass graft surgery was admitted to the hospital for an episode of right-sided chest pain, dyspnea, and presyncope. The episode occurred in the morning when the patient, while standing up from the bed and having a rightward rotation of the torso, experienced suddenly an intense right-sided chest pain perceived like “an explosion” and followed by moderate dyspnea at rest. Subsequently, while having intense chest pain, the patient became diaphoretic, severely lightheaded, then presyncopal and fell back into the bed. The chest pain persisted, worsened with the slightest movement, and was radiating from the right side to the left side of the chest.

His home medications included aspirin, valsartan, clonidine, furosemide and spironolactone combination (dose recently increased), bisoprolol, atorvastatin, and sublingual nitroglycerin.

### 2.2. Clinical Findings

Upon presentation to the emergency department the blood pressure was 140/90 mmHg, which dropped to 110/70 mmHg with orthostasis, and the pulse rate was 90 beats/min. His oxygen saturation was 86% in room air and 90% on 3 L/min of supplemental O_2_ via nasal cannula. On physical examination, jugular venous pressure was 5 cm H_2_O, breath sounds were diminished bilaterally especially on the right, with no rales. The heart sounds were regular, with no murmurs, rubs or gallops. His peripheral pulses were palpable with no peripheral edema.

### 2.3. Diagnostic Assessment and Therapeutic Intervention

**Figure 1:**
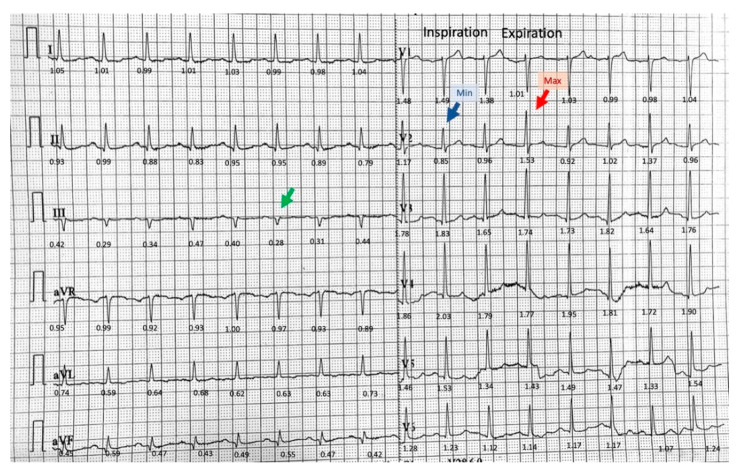
The electrocardiogram on admission. Voltage expressed in millivolts is displayed under each QRS complex. Phasic variations of the QRS amplitude seen in lead V2 with the minimum voltage (blue arrow) during inspiration and maximum voltage (red arrow) during expiration. Isolated low QRS voltage is seen in lead III (green arrow).

His laboratory workup revealed no elevation in serum troponin I or d-dimers, normal values of serum electrolytes, renal function tests, and blood count. Arterial blood gases on three liters per minute of oxygen via nasal canula were: pO_2_ 64.7 mmHg, pCO_2_ 33.3 mmHg, and pH 7.41. The electrocardiogram (ECG) showed sinus rhythm 96 beats/min, frontal plane QRS axis of 12°, PR 140 ms, QRS 92 ms, QTc 362 ms. There were marked phasic QRS voltage changes observed especially in lead V2 and lead III, with lower frequency modulation, which appeared to be timed with respiratory movements. The electrocardiogram also revealed isolated very low QRS voltage (<0.3 mV) in lead III (Figure 1 green arrow) [16].

**Figure 2:**
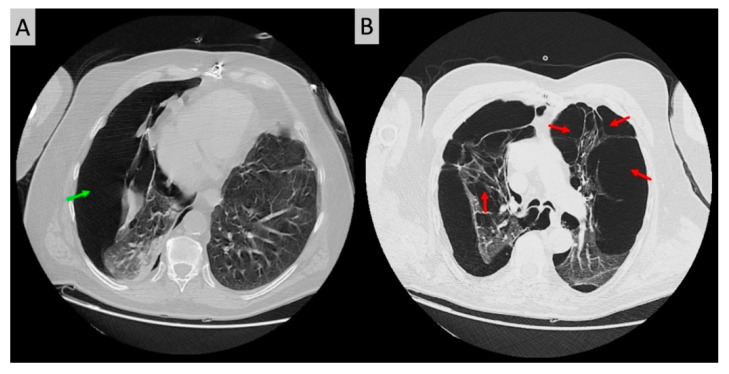
Transverse unenhanced chest CT scan revealing a large right-sided pneumothorax (green arrow) with compressive atelectasis of the right lung and emphysematous changes in both lungs (**A**); multiple bullae due to the underlying emphysema were noted in the left (red arrows) as well as the right lung in addition to the right-sided pneumothorax (**B**).

**Figure 3:**
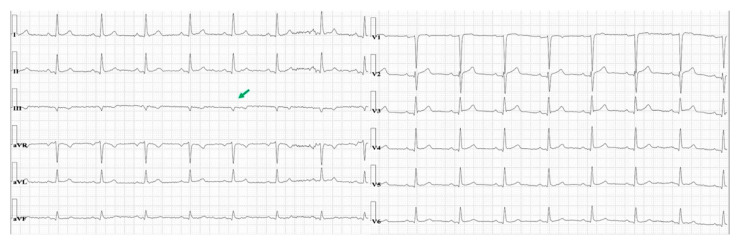
A 12-lead electrocardiogram recorded after chest tube placement. Isolated very low voltage is still seen in lead III (green arrow).

There were no acute ST-T abnormalities. While having recurrences of chest pain the patient experienced other episodes of lightheadedness and presyncope. The corresponding rhythm on the ECG monitor was sinus rhythm with no arrhythmia noted. Given the sudden onset chest pain and dyspnea, the patient underwent chest computed tomography, which revealed a large right-sided PTX, measuring 8 cm at the level of the hilum (Figure 2A). In addition, the structure of the lungs was abnormal, with bullous emphysema noted bilaterally (Figure 2B). Thoracic surgery was consulted urgently. A chest tube was inserted in the right chest under local anesthesia, and it was placed to suction. The patient experienced immediate symptomatic improvement. Phasic changes in the QRS amplitude noted on the initial electrocardiogram were significantly diminished after the chest tube was placed (Figure 3). To note also that the isolated very low voltage QRS complexes were present constantly in lead III. An echocardiogram revealed mild mitral regurgitation and left atrial enlargement, paradoxical septal motion consistent with prior coronary artery bypass surgery, and a left ventricular ejection fraction of 55%.

**Figure 4:**
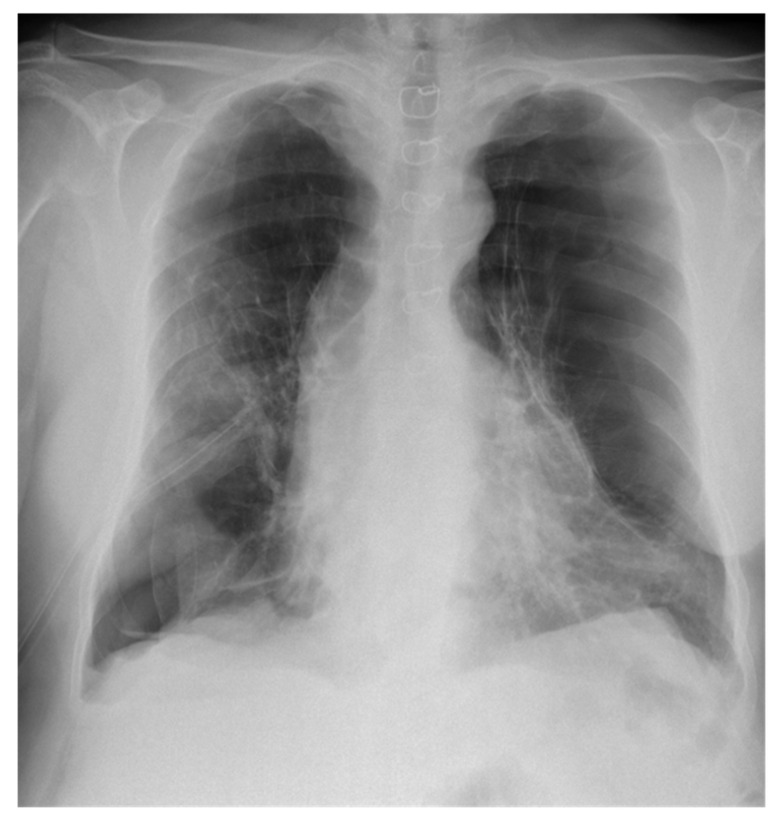
Posteroanterior chest X-ray showing limited expansion of the right lung day 4 after placement of the chest tube.

Over the following days, however, there was poor lung expansion confirmed on subsequent chest X-rays (Figure 4).

**Figure 5:**
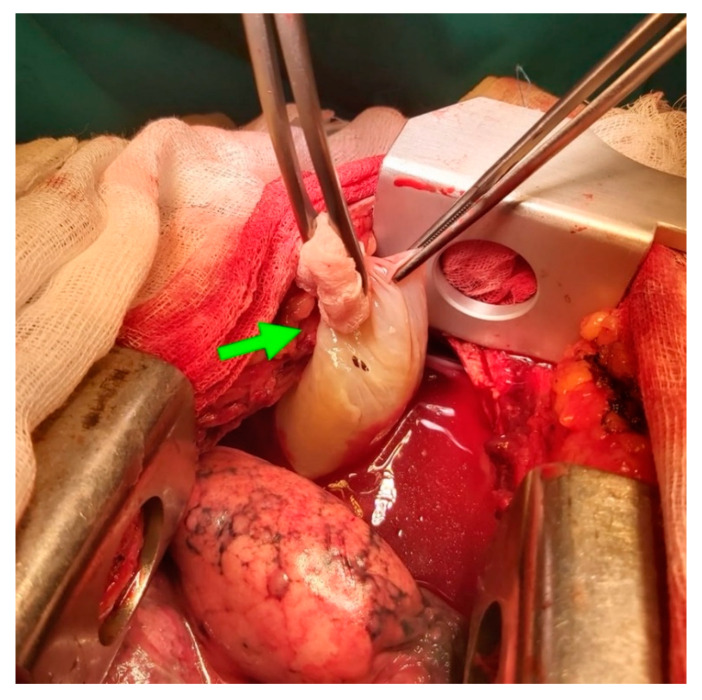
Intraoperatively a perforated bulla (green arrow) was found at the right middle lobe and was treated with bullectomy and talc pleurodesis.

The patient underwent open thoracic surgery. Intraoperatively, a perforated bulla was found at the right middle lobe and was thought to be the source of the PTX. Given this finding, bullectomy and talc pleurodesis was performed (Figure 5) [17]. Repeat imaging showed resolution of the PTX, and the patient was discharged home after several days of recovery.

### 2.4. Final Diagnosis

Given this presentation our diagnosis was: large right PTX caused by spontaneous rupture of an emphysematous bulla, and presyncope probably due to a vasovagal reflex.

## 3. Discussion

This is a case that displayed two unexpected ECG changes in a patient presenting with chest pain and presyncope.

### 3.1. Phasic Variations in the QRS Complex Amplitude

Previous cases of ECG alterations involving left-sided spontaneous PTX have been reported, encompassing different types of ECG changes: phasic voltage alternation of the QRS complex, low voltage QRS complexes, PR segment shifts, ST segment shifts, inverted T waves, loss of R waves in the precordial leads, and right axis deviation [7,8,9,10,11,12,13,14]. Among these changes the most consistently reported were the phasic QRS voltage changes in relationship with the respiratory movements. While most reports have described ECG changes in patients with left-sided PTX, there are a few reports of ECG alterations associated with right-sided PTX as well [15,18]. One possible mechanism behind the voltage alternation of the QRS complexes is the change in the position of the heart within the thoracic cavity with respiration [3]. This could lead to the registration of QRS complexes of variable voltage, reflecting the variable distance between the heart and the thoracic surface generated by the intrathoracic pressure alterations secondary to the PTX. Another possible hypothesis involves the interposition of the newly formed air cavity between the heart and the precordial electrodes from surface that could diminish the electrical conductance of the generated electrical potentials during inhalation, resulting a fixed pattern of QRS amplitude oscillation according to the breathing phases.

**Figure 6:**
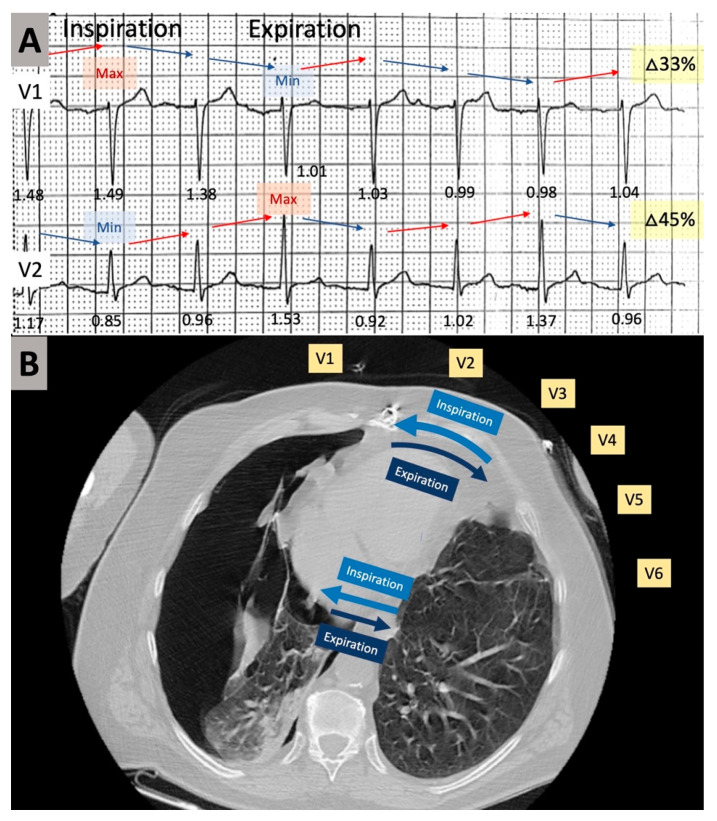
Possible mechanism for the phasic changes in QRS complex voltage noted on lead V1 and V2 on the initial 12-lead electrocardiogram. QRS voltages expressed in millivolts are shown under each beat. Both direction and magnitude of change of the QRS voltage are displayed. Red arrows point to an increase in voltage, while blue arrows depict a decrease in voltage. The complex with maximum and minimum voltage is depicted with “Max” and “Min” labels, respectively. The magnitude of change measured as percent change between the complex with minimum voltage and maximum voltage is shown on the right side of the tracing on a yellow label (**A**); A mechanistic hypothesis that could explain the phasic QRS complex changes. As a consequence of the atelectatic right lung, during inspiration (light blue arrows), the expansion of the left lung displaces the heart towards the right, bringing the septum and apex of the heart more towards lead V1 with an increase in QRS amplitude. During expiration the heart moves in the opposite direction (dark blue arrows). V1–V6 electrocardiographic leads across the precordium (**B**).

We have displayed in Figure 6 a hypothesis regarding the mechanism behind the phasic QRS amplitude changes in our patient. The heart had a relatively midline position, with a counterclockwise rotation, as displayed on the initial ECG, with a transition zone between V1 and V2. The large right-sided PTX caused significant atelectasis of the right lung. As a consequence of the atelectatic right lung, during inspiration, there was more air entering the left lung, displaced the heart towards the right, bringing the septum and apex of the heart more towards lead V1 with an increase in QRS amplitude during inspiration. Given the relatively midline positioned septum and apex in this counterclockwise rotated heart, the opposite was happening in lead V2, with a decrease of the QRS amplitude during the inspiratory effort. During expiration, due to the leftward shift of the apex and septum towards V2, the QRS voltage was decreasing in lead V1 and increasing in lead V2.

PTX can occur spontaneously or can be a complication of different procedures including electrophysiologic procedures like implantation of cardiac implantable electronic devices. The decision to place a chest tube is usually made based on symptoms and signs like dyspnea, hypotension, diminished breath sounds on the affected side distended neck veins, in conjunction with other variables like respiratory rate and oxygen saturation [19]. The presence of phasic variations in QRS amplitude, which has only reported cases with a large PTX, could have additional value when making the decision to place a chest tube.

### 3.2. Isolated Very Low Amplitude QRS Complex in Frontal Leads

The exact mechanism of the presyncopal episode in our patient is not known. However, given the onset during intense pain, the association with diaphoresis, occurrence in a patient with a degree of intravascular volume depletion in the setting of diuretic therapy suggests a reflex mechanism. A degree of reduction of the venous return secondary to the compression caused by the PTX might have played a role as well. An ECG finding recently found to occur in patients with reflex syncope, and to be associated with positivity of tilt table testing as well as risk of syncope recurrence is the presence of isolated very low voltage in the frontal leads [16,20,21]. Our patient has isolated very low amplitude (<0.3 mV) QRS complexes in lead III before and after chest tube placement (Figure 1 and Figure 3).

### 3.3. Limitations

Given that the rarity of case reports and the lack of original research studies on ECG changes in right-sided pneumothorax it is difficult to assess the diagnostic accuracy of these ECG abnormalities.

### 3.4. Summary and Future Perspectives

Reports of ECG changes in PTX, like the one presented here, are extremely rare. This may be due to the rarity of such occurrence, or, more likely is related to the lack of awareness about ECG abnormalities in such patients. This case presentation could represent a stimulus for more research in this field.

## 4. Conclusions

We have described a case of a right-sided pneumothorax associated with presyncope presenting with phasic changes in the QRS amplitude in the precordial leads. Such changes, when present in a patient with sudden onset chest pain and dyspnea, should prompt a diagnostic workup for possible pneumothorax. The workup for PTX needs to be done urgently since the ECG changes seem to be associated with large air collections with potential for hemodynamic deterioration

## Data Availability

Not applicable.
